# Infectious Salmon Anemia Virus Shedding from Infected Atlantic Salmon (*Salmo salar* L.)—Application of a Droplet Digital PCR Assay for Virus Quantification in Seawater

**DOI:** 10.3390/v13091770

**Published:** 2021-09-04

**Authors:** Simon Chioma Weli, Lisa-Victoria Bernhardt, Lars Qviller, Ole Bendik Dale, Atle Lillehaug

**Affiliations:** Norwegian Veterinary Institute, P.O. Box 64, N-1431 Ås, Norway; Lisa-Victoria.Bernhardt@vetinst.no (L.-V.B.); Lars.Qviller@vetinst.no (L.Q.); ole.b.dale@vetinst.no (O.B.D.); atle.lillehaug@vetinst.no (A.L.)

**Keywords:** ISAV concentration, membrane filter, ISAV detection, ISAV transmission, anemia, aquaculture, fish virus

## Abstract

*Infectious salmon anemia virus* (ISAV) infection is currently detected by fish sampling for PCR and immunohistochemistry analysis. As an alternative to sampling fish, we evaluated two different membrane filters in combination with four buffers for elution, concentration, and detection of ISAV in seawater, during a bath challenge of Atlantic salmon (*Salmo salar* L.) post-smolts with high and low concentrations of ISAV. Transmission of ISAV in the bath challenge was confirmed by a high mortality, clinical signs associated with ISA disease, and detection of ISAV RNA in organ tissues and seawater samples. The electronegatively charged filter, combined with lysis buffer, gave significantly higher ISAV RNA detection by droplet digital PCR from seawater (5.6 × 10^4^ ISAV RNA copies/L; *p <* 0.001). Viral shedding in seawater was first detected at two days post-challenge and peaked on day 11 post-challenge, one day before mortalities started in fish challenged with high dose ISAV, demonstrating that a large viral shedding event occurs before death. These data provide important information for ISAV shedding that is relevant for the development of improved surveillance tools based on water samples, transmission models, and management of ISA.

## 1. Introduction

Infectious salmon anemia virus (ISAV) is a water-borne virus that causes infectious salmon anemia (ISA) disease in Atlantic salmon (*Salmo salar* L.) [1,2,3,4]. ISAV, which belongs to the genus *Isavirus* of the family *Orthomyxoviridae,* exists as a putative non-virulent variant, with a full-length highly polymorphic region (HPR) [5,6,7,8,9] called HPR0 ISAV, and the highly pathogenic ISAV variants with over 30-different HPR-deleted subtypes [10,11,12]. The highly pathogenic ISAV variants are thought to arise from genetic deletion by mutation (HPR-deleted) in non-virulent strains (HPR0-strains) [8].

HPR0 ISAV does not give clinical disease, but causes a short, transient gill epithelial infection [13]. The HPR-deleted ISAV causes generalized and lethal disease which is characterized by anemia, circulatory disturbances, and bleedings in several organs of the fish [14], with cumulative mortality in infected marine fish populations varying from 0 to 90% [15]. Since the major ISA epidemic in Norway in the 1990s, ISAV remains a serious fish pathogen, probably because of asymptomatic infections in wild and farmed Atlantic salmon with HPR0, and the potential for emergence of new virulent strains [8]. In Norway, from one to 20 ISA outbreaks occur annually, with most of the outbreaks clustering in northern Norway during past years [16].

Virulent ISAV primarily infects and multiplies within the host-fish, and virus particles are shed into the seawater environment, causing a risk of spread within the population and to other populations in the neighborhood. Phylogenetic analyses of virus isolates from disease outbreaks indicate that the infection spreads from a primary outbreak and causes secondary outbreaks with the same HPR-deleted subtype due to horizontal transmission to other farm sites [1,2,3,4,17,18]. Nearly half (43%) of ISA outbreaks in Norway from 2004 to 2009 were caused by the spread between farms in the proximity [1]. Thus, surveillance of ISAV in seawater can be an alternative approach to current surveillance methods based on detection of virus in clinically diseased or healthy sampled fish, which is currently implemented in zones with ISA-outbreaks in farmed Atlantic salmon populations. This will require the development of a method for concentration and early detection of ISAV shedding that will facilitate effective virus surveillance and enable earlier implementation of disease control measures.

The current laboratory methods used for diagnosis of ISA and detection of ISAV are largely dependent on fish sampling for nucleic acid isolation and amplification, histopathology, and immunohistochemistry (IHC) analysis [14]. Fish sampling, in general, is selective, resource demanding, time consuming, and in many cases, could be problematic from an animal welfare point of view. In addition, when there is a low prevalence of ISAV in the farm, detection of ISAV from individual fish can be difficult. However, in the aquatic environment, the virus is probably spread by water via sea currents, therefore it may be easier to detect it in water samples.

Recently, we have developed a method for detection of *Salmonid alphavirus* (SAV) in seawater for surveillance purposes [19,20]. In order to also implement this methodology for ISAV, in vitro experiments for concentrating virus in seawater have been carried out [20]. The study involved spiking of ISAV in seawater, followed by concentration of virus by adsorption to charged membrane filters and virus elution, before molecular detection. Here, we extended the investigations from the in vitro study to an experimental (ISAV) bath challenge trial with Atlantic salmon. The aim of this study was to evaluate the different membrane filters and buffers for concentration of ISAV directly from seawater in tanks holding infected fish, followed by virus detection by a reverse transcriptase droplet digital PCR (RT-ddPCR) assay.

## 2. Materials and Methods

### 2.1. Virus and Cell Cultures

The ISAV-Glesvær isolate [21] was propagated in an ASK cell line (ATCC^®^ CRL-2747™) as previously described [22]. Briefly, cells were grown at 20 °C in T-150 culture flasks containing Leibowitz L-15 medium (Thermo Fisher Scientific, Warrington, United Kingdom) supplemented with 10% fetal bovine serum (FBS) and gentamicin (Lonza, USA). The ISAV isolate was used to inoculate the cell culture and incubated for seven days at 15 °C. Cell culture supernatant was harvested and clarified by centrifugation at 3800 g for 10 min at 4 °C. The clarified supernatant in 1 mL aliquots was frozen at −80 °C. Virus titer was calculated according to the 50% end-point method of Reed and Muench (1938) [23], and expressed as the dilution of the virus causing 50% tissue infection per milliliter (TCID_50_/mL).

### 2.2. Fish

The fish used in this study were Atlantic salmon post-smolts (mean weight 80 g), which were obtained from Stofnfiskur Iceland (SF Optimal) and reared at the Industrial and Aquatic Laboratory fish facility (ILAB, Bergen High Technology Centre, Bergen, Norway). The fish were unvaccinated and pre-screened at 5 g and 15 g of size, and tested negative for (SAV), (ISAV), *Infectious pancreatic necrosis virus* (IPNV), *Piscine myocarditis virus* (PMCV), *Piscine orthoreovirus* (PRV), and *Salmon gill poxvirus* (SGPV). Parent fish were pre-screened for the same viruses, apart from SGPV. The fish were maintained in tanks supplied with filtered (20 µm), UV-treated seawater originating from 105 m depth, from Byfjorden (Norway), with a salinity of 34‰. A constant temperature of 12 °C, a daily photoperiod of 12:12 h light and dark, and oxygen saturation of about 80%, were maintained throughout the challenge period.

### 2.3. Experimental ISAV Bath Challenge

**Figure 1:**
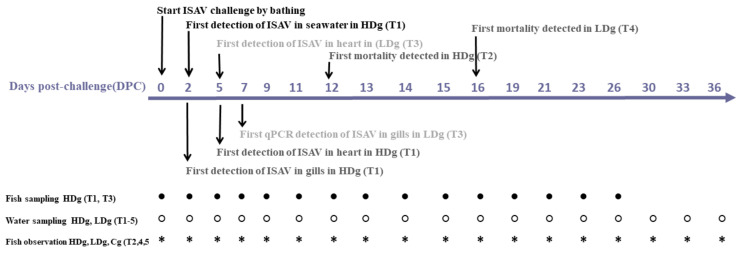
Schematic diagram of the experimental setup and summary of the results of infectious salmon anemia virus bath challenge trial with post-smolt Atlantic salmon. At day 0, fish in three different tanks were bath challenged with 2 × 10^4^ TCID_50_/mL ISAV (high dose group; HDg), 2 × 10^2^ TCID_50_/mL ISAV (low dose group; LDg), or virus-free L-15 cell culture medium (negative control; Cg). After 2 h bath challenge, fish from the three different challenge groups were distributed into five identical 500 L seawater tanks (T1–T5) as follows: high dose group (HDg) were transferred to sampling tank (T1) and observational tank (T2) respectively, low dose group (LDg) were transferred into sampling tank (T3) and observational tank (T4), while the negative control group (Cg) was transferred into tank (T5). At different days post challenge (DPC), seawater and fish samples were collected from the different groups.

The bath challenge trial with ISAV in seawater was performed as shown in Figure 1. Briefly, 300 fish were divided between three 200-L tanks containing seawater and bathed for 2 hr, during which the water flow was stopped. Group 1: 120 fish were bathed with 2 × 10^4^ TCID_50_/mL ISAV (designated as high dose group; HDg); group 2: 120 fish were bathed with 2 × 10^2^ TCID_50_/mL ISAV (designated as low dose group; LDg); and group 3: 60 fish were bathed with a dilution of virus-free Leibovitz-15 (L-15) cell culture medium containing 2% FBS (designated as negative control group; Cg). The tanks were continually aerated, and the oxygen levels were closely monitored. After 2 h, fish from the three different challenge groups were transferred to five identical 500 L seawater tanks (T1–T5) as follows: sixty fish from group 1, designated as high dose group, were transferred to each of the sampling and observational tank, T1 and T2, respectively. Sixty fish from group 2, designated as low dose group, were also transferred to each of the sampling and observational tank, T3 and T4. The sixty fish from group 3, designated as negative control group, were transferred to tank T5. All tanks were monitored for an hour after the fish were transferred to ensure welfare of the fish. The water flow rate in all tanks was constant throughout the experiment, with an average flow rate of 950 L/h/tank and which was set according to the biomass, dissolved oxygen level, and water temperature, in order to meet optimal oxygen levels for the fish. The challenge experiment lasted for 36 days, and the daily fish survival was recorded. All animal procedures were approved by the Norwegian Food Safety Authority based on the guidelines of the Norwegian Animal Welfare Act.

### 2.4. Fish Sampling

Fish sampling was performed at 15 different time points: 0, 2, 5, 7, 9, 11, 12, 13, 14, 15, 16, 19, 23, 23, and 26 days post-challenge (DPC). A total of seven fish, three fish from each of HDg (tank-T1) and LDg_,_ (tank-T2) and one fish from Cg (tank-T5) were randomly sampled from the sampling tanks at each time point and anesthetized in Finquel^®^ vet. 1000 mg (100 mg/L). Once immobilized, the fish were euthanized, gross pathology was evaluated, and tissue samples were aseptically collected for real-time PCR (qPCR), histopathology and IHC. For qPCR analysis, gill and heart tissue samples were collected in RNAlater^®^ (Qiagen, Hilden, Germany), kept at 4 °C overnight and stored at −80 °C prior to RNA extraction. Samples for histopathology and IHC were collected in 10% buffered formalin.

### 2.5. Immunohistochemistry

Immunohistochemistry (IHC) for ISAV was performed as previously described [24]. Briefly, sections of formalin-fixed paraffin-embedded tissue were prepared on poly-lysine-coated slides, dewaxed, and subjected to microwave oven treatment. Rabbit antibody to recombinant nucleoprotein ISAV (ISAV-NP) [25] was used for virus detection. The Vectastain ABC-AP kit (Vectastain anti-rabbit Ig ABC-AP kit; AK 5001; Vector Laboratories, Inc.) was used for detection of bound antibody employing Fast Red (1 mg mL) and naphthol AS-MX phosphate (0.2 mg mL) with 1 mM levamisole in 0.1 M Tris-buffered saline (TBS) (pH 8.2) as the substrate.

### 2.6. Water Sampling and Concentration of ISAV in Water

Water sampling was performed at the same time points as fish sampling, as well as at 30, 33, and 36 days post-challenge (DPC). At each time point, one L of seawater was sampled from each of the tanks T1–T5. The concentration of ISAV from 1 L seawater samples from each of the five seawater tanks was performed as described previously [26]. During preliminary experiments, two different filters with different buffer combinations were evaluated and method A–E was chosen for this study (see below). Since the water retention of MF^-^ filter is different from the MD^+^ filter, two different elution volumes were applied (2.4 mL or 4.0 mL), to get the best possible solution to recover 1 mL concentrates after elution of adsorbed virus from the filters. Briefly, the electronegatively charged nitrocellulose MF hydrophilic membrane filter (MF^−^, 47 mm diameter and 0.45-µm pore size) (Millipore, USA) and the electropositively charged 1 MDS Zeta Plus^®^ Virosorb^®^ membrane filter (MD^+^, 47 mm diameter) (CUNO Incorporated, Meriden, CT 06450, USA) were used to adsorb the ISAV particles from the seawater samples during the concentration process. To elute the adsorbed virus particles from the membrane filters, five different concentration methods with different combinations of filters and buffers were used:

Method A—MF^−^/buffer 1 method (NucliSENS^®^easyMAG ^®^lysis buffer (bioMérieux SA, France).

Method B—MD^+^/buffer 1 method (NucliSENS^®^easyMAG ^®^ lysis buffer (bioMérieux SA, France).

Method C—MF^−^/buffer 2 method (1 mM NaOH pH 9.5 buffer)

Method D—MF^−^/buffer 3 method (Leibowitz L-15 medium, Life Technologies, UK) + 2% FBS (pH 9.0).

Method E—MD^+^/buffer 4 method (Leibowitz L-15 medium) + 2% FBS (pH 7.5).

For the MF^−^/buffer 1 method, the filter was immediately placed into a Petri dish containing 2.4 mL of the buffer and shaken for 30 min at 600 RPM, in room temperature. For elution with MD^+^ buffer 1 and buffer 2, 3, and 4 methods, each filter was cut into small pieces and placed in a 50 mL Falcon tube containing 4 mL of the respective buffer. Each sample was vortexed 3 × 1 min, with 5 min rest intervals at room temperature, and concentrates were stored at −80 °C prior to RNA extraction and ISAV detection by RT-ddPCR. Retrospective (back) calculations were used to calculate ISAV copy numbers in 4 or 2.4 mL seawater concentrates by taking into consideration that ISAV was concentrated from 1 L of seawater, followed by elution in either 4 or 2.4 mL buffer, from which 1 mL was used to extract RNA. The extracted RNA was eluted in 50 µL, and 1.8 µL of RNA was used for RT-ddPCR assay.

### 2.7. RNA Extraction of Organ Tissue

Total RNA was extracted from gill and heart tissue according to generic QIAcube protocol (Qiagen^®^, Hilden, Germany). RNA was eluted in 200 µL of elution buffer and stored at −80 °C prior to use in RT-qPCR.

### 2.8. RNA Extraction of Concentrated Seawater Samples

Total RNA was extracted from 1 mL from all concentrated seawater samples according to the generic easyMAG protocol (bioMèrieux, Marcy l’Etoile, France). Briefly, 1 mL of NucliSENS^®^ easyMAG^®^ Lysis Buffer (bioMérieux, Marcy l’Etoile, France) was added to 1 mL of seawater concentrate, followed by RNA extraction. RNA was eluted in 50 µL of elution buffer and stored at −80 °C prior to use in RT-ddPCR.

### 2.9. Real–Time Quantitative PCR (RT-qPCR)

The RT-qPCR was used for ISAV detection in fish tissue samples. The primers F-primer: 5′-CTACACAGCAGGATGCAGATGT-3′, R-primer: 5′-CAGGATGCCGGAAGTCGAT-3′, and probe: 5′-6FAM-CATCGTCGCTGCAGTTC -MGBNFQ-3′ were used for ISAV detection [27]. All qPCR analyses were performed on Stratagene Mx3000P qPCR System (Agilent Technologies LTD, Cheshire, United Kingdom ) using Brilliant III Ultra-Fast QRT-PCR kit (cat. no. 600884, Agilent Technologies) set-up: 55 °C for 10 min; 95 °C for 3 min, and then 45 cycles with 95 °C for 10 s and 60 °C for 20 s. For each tissue sample, the ISAV RT-qPCR assay was performed in duplicates, as a 20-μL reaction containing 2 μL of extracted RNA, 10 μL 2× Brilliant III Ultra III buffer, 1 μL RT/RNase block, 0.3 µL Ref. dye (Brilliant III Ultra-Fast QRT-PCR Master Mix, Agilent Technologies) and probe/primers at a final concentration of 500 nM for forward- and reverse primers, and 300 nM for the TaqMan probe.

### 2.10. Reverse Transcriptase Digital Droplet PCR (RT-ddPCR)

The RT-ddPCR was used for detection of ISAV in filtered water concentrates. The same primer and probe used for RT-qPCR assays were used for RT-ddPCR. The annealing temperature and primer/probe concentrations have been optimized as described previously [20]. The RT-ddPCR was performed in 20 μL volume, with 1.8 μL of RNA extracted from the seawater concentrates, and with primers and probe at final concentrations of 900 nM and 250 nM, respectively. A one-step RT-ddPCR advanced kit master mix (Bio-Rad) was used, and PCR was performed in a C1000 Touch thermal cycler (Bio-rad) with a 96- deep well reaction module. The temperature profile for PCR was 60 °C for 60 min, 95 °C for 10 min, followed by 45 cycles of 95 °C for 30 s, from 50.0 to 63.0 °C annealing for 1 min, and a final 98 °C for 10 min. For all step a ramp rate of 2.5 °C /min was used. Results were analyzed with Quantasoft 1.7.4.0917 software (Bio-Rad). The threshold for distinguishing positive from negative droplets was set manually.

### 2.11. Statistics

In order to compare the performance of the five different concentration methods (the combinations of filter and buffer), and of the buffers and filters separately, we performed pairwise comparisons between the concentration methods, the two filters, and the four buffers. The comparisons were performed with a post hoc Tukey-HSD-tests based on two fitted generalized linear models (GLM). A GLM is a flexible generalization of the linear regression model, allowing the response variable to have other error distributions than the normal distribution. Initial analyses revealed that the errors in the response followed the Poisson distribution. The Poisson GLM is a regression analysis where the natural logarithm of the model predictions is a linear function of the explanatory variables, and where the variance is equal to the expected value. The post hoc Tukey-HSD-test is a way to compare the levels of a factor, without inflating the type I error rate when performing multiple hypotheses tests. First, we fitted two GLM models with virus concentration in water as the response, to assess the unbiased comparison of the concentration methods, and filter and the buffer. In the first model, we included the concentration method as an explanatory variable. In the second model, we treated filter and buffer as two separate explanatory variables.

The disease and viral shedding in the experimental tanks developed over time, such that viral concentrations at time point *t* may depend on the concentrations at time point *t-1.* We therefore suspected a time series effect with temporal autocorrelation between the observations, reflecting the development of the infection in the individuals. This was handled by testing days post challenge (DPC) as a non-linear effect, using both spline functions and polynomials during the model selection. We also tested for interaction effects between DPC and infectious dose (HDg and LDg) to allow for different development in disease, depending on the infectious dose they were challenged with. In addition, we compared the copy numbers from water samples with *Cq*-values from the tissue samples, following a similar GLM-based framework with a Poisson error distribution. All model selections were performed using the Akaike information criterion (AIC), and model critique was performed using cross-validation with five data points as validation data. In addition, we did a manual examination of the residuals. All statistical analyses were performed using the R statistical software [28]. The Tukey-HSD from a GLM was performed using the multcomp package in R [29], and dispersion in the residuals was evaluated using the dispersion test function in the AER package in R statistical software (version 3.6.2) [30].

## 3. Results

### 3.1. ISAV Bath Challenge and Fish Survival

**Figure 2:**
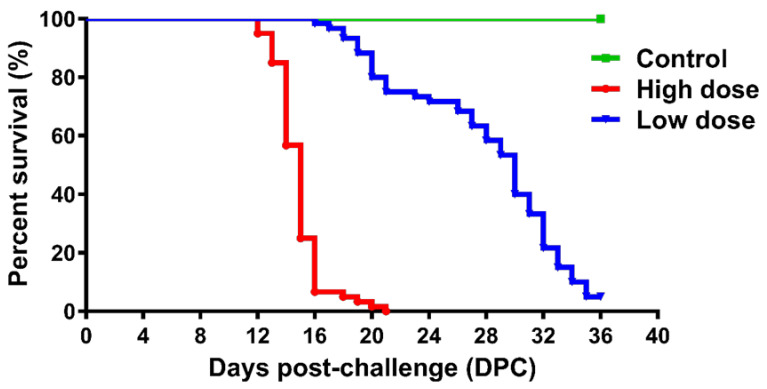
Kaplan-Meier survival plot of fish challenged with 2 × 10^2^ TCID_50_/mL ISAV (high dose group; HDg), 2 × 10^4^ TCID_50_/mL ISAV (low dose group; LDg) or virus-free L-15 cell culture medium (negative control; Cg) in the three observational tanks (T2: high viral dose tank; T4: low viral dose tank; and T5: negative control tank).

Transmission of ISAV to the fish during bath challenge was confirmed by high mortality, clinical signs, and detection of ISAV RNA in fish and water samples. In the observational tanks, which comprise one high dose virus group (T2) and one low dose virus group (T4) that were used to monitor survival, fish mortality started at 12 DPC in HDg ISAV exposed individuals and peaked at 21 DPC, with a cumulative mortality of 100%, as shown in Figure 2. Mortality in the LDg ISAV exposed individuals commenced at 16 DPC, and it increased at a slower pace, reaching a mortality of 95% at 36 DPC (Figure 2). In the sampling tanks, which comprise one high dose virus group (T1) and one low dose virus group (T3), the percentage mortality rates were 100% and 95%, respectively. No mortality or moribund fish were observed in the negative control fish.

### 3.2. Autopsy and Clinical Signs

**Figure 3:**
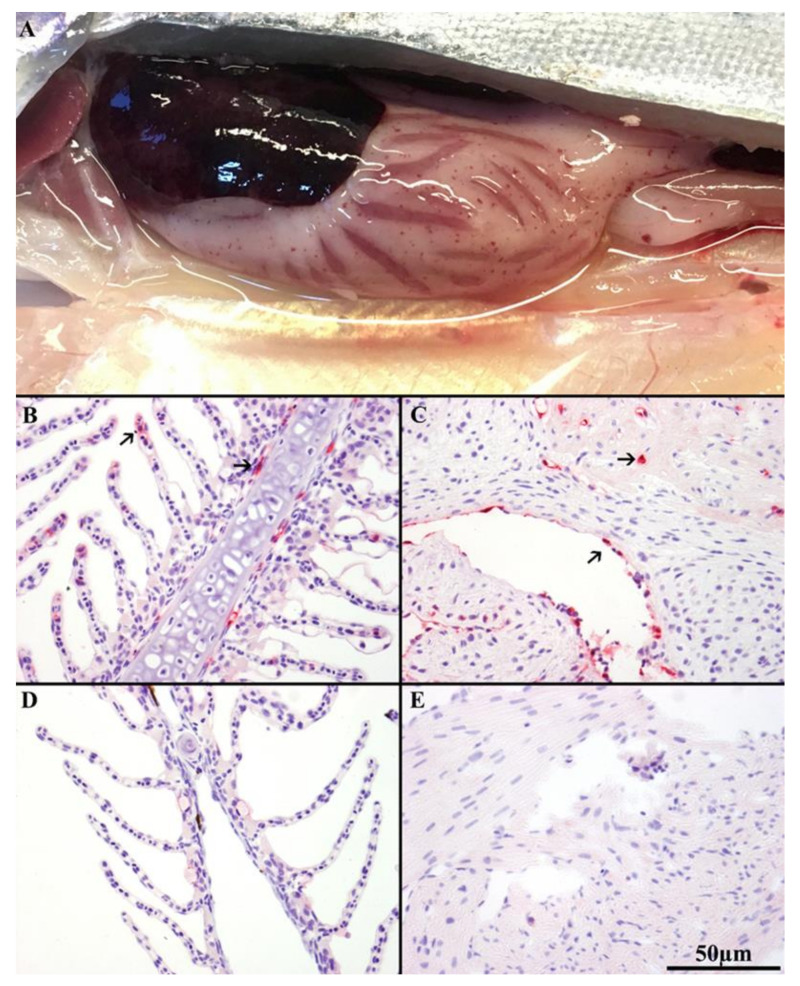
(**A**) Autopsy showing visible internal clinical ISA disease signs such as liver discoloration, enlarged spleen, ascites, and petechial hemorrhaging of the visceral fat in HDg ISAV exposed individuals at 9DPC. Immunohistochemistry (IHC) of ISAV-infected Atlantic salmon (*Salmo salar* L.) with anti-ISAV NP rabbit antibody. (**B**) Histology of gill of infected LDg fish sampled at 9DPC, showing strong positive staining (arrows) of epithelial cells of interior lamellae, endothelial cells, and pillar cells. (**C**) Histology of heart of clinical HDg fish at 9DPC showing strong positive staining (arrows) of endothelial cells covering the spongious heart muscle and the compact heart muscle. (**D**,**E**) show negative staining of the gill and heart tissues from the sampled negative control fish at 9DPC.

Affected fish exhibited external and internal ISA signs as described previously [31]. Initial signs of disease onset in HDg ISAV exposed individuals were observed at 9 DPC, including change in behavioral patterns, such as swimming near the surface of the tank, loss of balance, lethargy, and lack of response to stimuli. Internal pathological findings, such as liver discoloration, enlarged spleen, ascites, petechial hemorrhaging of the visceral fat, were visible in some individual fish from HDg at 9 DPC, as shown in Figure 3A, and fish from LDg at 16 DPC, respectively. Individuals from the control group showed no clinical signs associated with ISA. Histological examination of gills from individual fish from HDg and LDg revealed focal, moderate hypertrophy, and hyperplasia of the lamellar epithelium (data not shown).

### 3.3. Immunohistochemistry (IHC)

IHC staining was performed on 30 HDg, 45 LDg, and 15 Cg individual fish tissues. Results from the IHC showed specific staining in potential target cells, including epithelial cells of interior lamellae, pillar cells, and endothelial cells of the gills (Figure 3B), and the endothelial cells covering the spongious-heart muscle and the compact heart muscle (Figure 3C). Positive labelling was found in 9 of 30 (30%) gill and in 14 of 30 (46.6%) heart tissues of the HDg fish, compared to 12 of 45 (26%) and 18 of 45 (40%) in the gills and heart tissues in the LDg of individual fish, respectively. The epithelial and endothelial cells of the gills from the sampled fish individuals from HDg and LDg were positive by IHC before any of the other organ tissues, and appeared to be the first tissue in which viral replication was initiated. As the infection progressed, from 9 to 16 DPC, the heart had increased numbers of positive endothelial cells. ISAV positive staining in the gill and heart were observed first in individual fish from HDg before those from LDg. No positive staining was observed in the gills or heart tissues from the negative control fish collected from Cg, as shown in Figure 3D,E.

### 3.4. Detection of ISAV in Fish Tissue Samples

**Figure 4:**
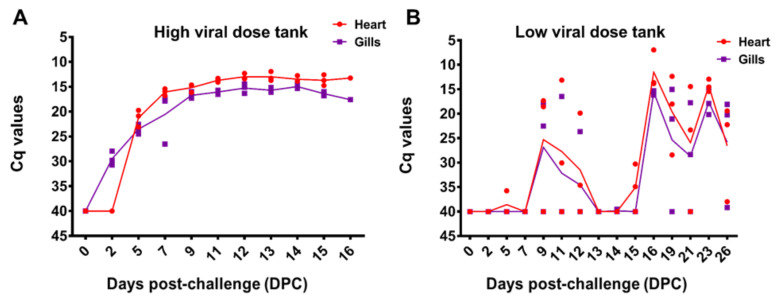
Infectious salmon anemia virus infection levels in the gills and heart tissues sampled at different time points (DPC) in HDg and LDg ISAV exposed individuals (**A**). The concentration of ISAV RNA per fish were analyzed by RT-PCR and presented as quantification cycle (Cq) values per days post-challenge (**B**). The dots represent the observed data from individual fish, while the lines represent the mean of the fish at each sampling time point.

Three fish tissue samples from each sampling time points from the bath challenge trial were analyzed from each of the parallel tanks of HDg (tanks T1 and T2) and LDg (tanks T3-T4) and also from tank Cg (T5). The results, shown in Figure 4, showed there was a delayed infection in the LDg compared to HDg ISAV exposed individuals. The peak of infection in gills and heart occurred at 16 DPC in the LDg fish, as opposed to the HDg fish, with result showing approximately the same levels ISAV RNA at 9–16. The first detection of ISAV in gill and heart in the HDg ISAV exposed individuals was made at 2 and 5 DPC, whereas, in the LDg ISAV exposed individuals, ISAV was detected at 5 and 7 DPC, respectively, as shown in Figure 4A,B. HDg ISAV exposed individuals also showed higher viral RNA. At the peak of infection, fish mortalities were also high, which indicated a relationship between ISAV and the mortality levels in the ISAV exposed individual fish. No ISAV was detected in fish tissues from Cg and those from the pre-challenge sample taken before bath challenge. There were not enough data to compare ISAV concentrations recovered from the gill and heart samples with the recovery from the water samples.

### 3.5. Detection of ISAV in Seawater Samples

One L seawater samples were collected at each sampling time points from each of the parallel tanks HDg (tanks T1 and T2) and LDg (T3 and T4) and from tank Cg (T5). However, only seawater samples collected from tanks (T1-HDg, T3-LDg and T5-Cg), the same tanks where fish sampling was performed, were analyzed by RT-ddPCR, in order to compare the results from seawater with the fish tissue samples. ISAV RNA in seawater samples were measured using a RT-ddPCR assay targeting segment 8 of the ISAV genome. In fish tanks challenged with high (HDg) and low dose (LDg) ISAV, viral shedding in seawater was first detected at 2 DPC (5 × 10^3^ ISAV RNA copies/L) and (32 ISAV RNA copies/L) respectively. Virus shedding in water peaked at 11 DPC (2.7 × 10^5^), 1 day before mortalities in HDg fish and at 12 DPC (1.6 × 10^5^), 5 days before mortalities in LDg fish. The average ISAV RNA concentration in the seawater from the HDg was higher than those of the LDg fish Figure 4. ISAV was not detected in the negative control tank (Cg).

### 3.6. Comparison of ISAV Recovery from the Five Concentration Methods

**Figure 5:**
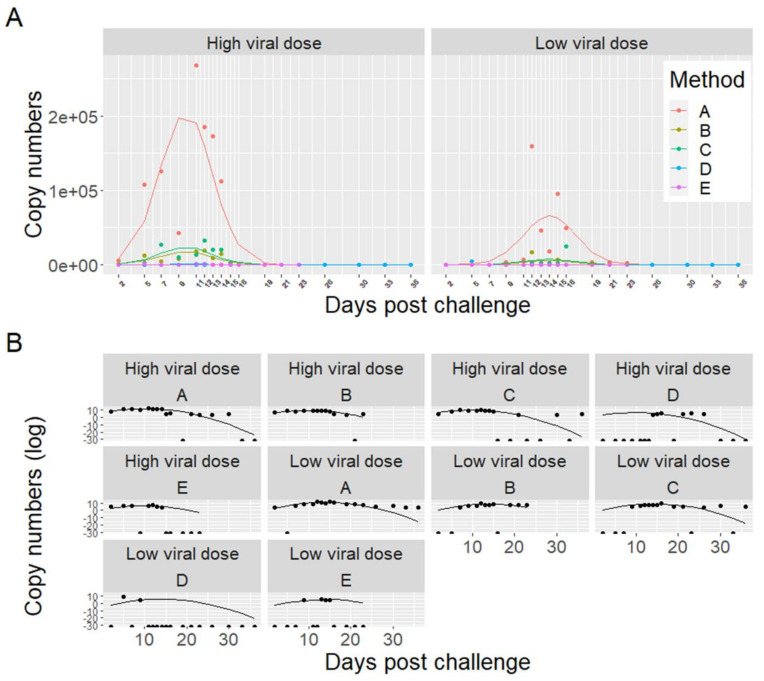
Comparison of five different concentration methods (**A**–**E**) for detection of infectious salmon anemia virus RNA from 1 L seawater collected from high viral dose (HDg) and low viral dose (LDg) ISAV exposed tanks. ISAV RNA in seawater samples were measured using a RT-ddPCR assay which targeted segment 8 of the ISAV genome. The dots represent the observed data, while the lines represent the model predictions. Panel A illustrates the untransformed data. Panel B illustrates each method and dose separately with copy numbers transformed with the natural logarithm to better illustrate the data points and model fit. Note that taking the logarithm of zero returns negative infinity. This is illustrated with half points at the bottom of the graphs in (**B**).

**Figure 6:**
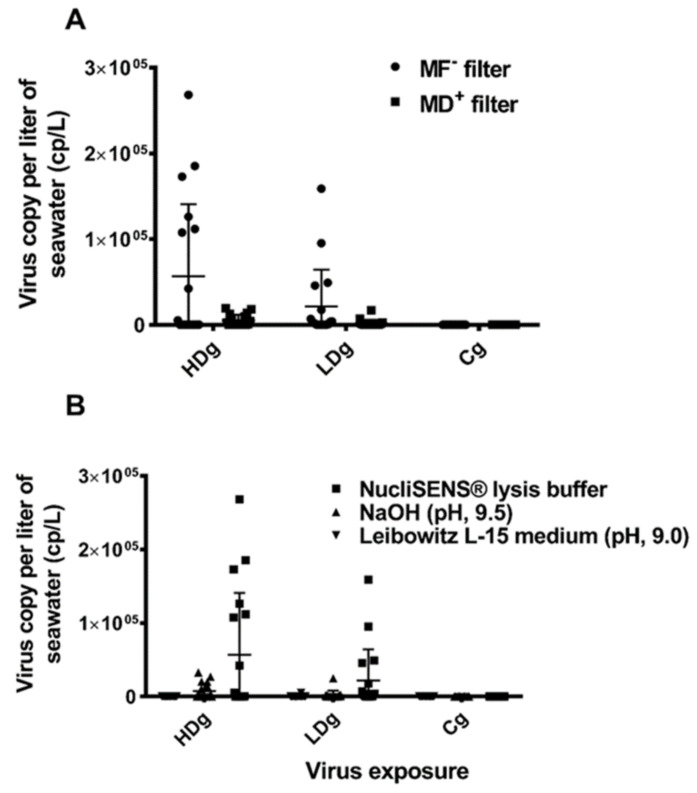
Comparison of the performance of the different buffers and filters used in concentration and detection of infectious salmon anemia virus RNA from 1 L seawater collected from HDg, LDg ISAV exposed individuals, and negative control Cg individual. (**A**) One liter of seawater was collected from HDg, LDg ISAV exposed tanks and negative control Cg tanks was concentrated with electronegative charged nitrocellulose MF hydrophilic membrane filter and electropositive charged 1 MDS Zeta Plus^®^ Virosorb^®^ membrane filter. Samples were eluted with NucliSENS^®^ lysis buffer and ISAV RNA in the seawater sample was measured by RT-ddPCR. Bars show the average detection at different time points (DPC), *n* = 18. (**B**) One liter of seawater was collected from HDg, LDg ISAV exposed tanks, and negative control Cg tanks were concentrated with electronegative charged nitrocellulose. MF hydrophilic membrane filter was eluted with NucliSENS^®^ lysis buffer, 1 mM NaOH pH 9.5 buffer, and L-15 medium + 2% FBS (pH 9.0), respectively. ISAV RNA in seawater samples was measured by RT-ddPCR. Bars show the average detection at different time points (DPC), *n* = 18.

Model selection revealed that there were significant differences in recovery between the concentration methods. The development of viral shedding followed a second order polynomial, and a significant interaction with viral dose indicated that the shedding followed a different progression between the viral dose groups (HDg and LDg). The model is presented graphically in Figure 5, showing the performances of the five concentration methods (A–E) for detection of ISAV RNA from seawater samples collected from the HDg and LDg ISAV exposed individuals and Cg. There were significant differences in ISAV RNA recovery with different methods (Table 1). In both HDg and LDg ISAV exposed individuals, the amount of ISAV RNA detected with the MF^-^/buffer 1 method (A) was higher compared to the other four concentration methods. The method A recovered an average of 5.6 × 10^4^ ISAV RNA copies/L compared to the MD^+^/buffer 1 method (B) 6.4 × 10^3^ ISAV RNA copies/L, method C 7.1 × 10^3^ ISAV RNA copies/L, method D 59 ISAV RNA copies/L, and method E which recovered 2.2 × 10^2^ ISAV RNA copies/L, throughout the challenge (Figure 5). Furthermore, comparison of the two different filters and four different buffers showed that the electronegative membrane filter and NucliSENS^®^ easyMAG^®^ buffer performed best in ISAV concentration and detection by RT-ddPCR (Figure 6A,B, Table 1). The increase in ISAV infection levels (Figure 4) was observed to appear at the same time as the increase in recovered ISAV RNA copies in the seawater samples (Figure 5). There were not enough data, however, to separate the effects from time series autocorrelation and a possible correlation between PCR results in fish and seawater samples.

## 4. Discussion

In this study, we have demonstrated that infectious salmon anemia virus (ISAV) can be detected from seawater samples collected from tanks holding Atlantic salmon (*Salmo salar* L.) post-smolts, which were experimentally infected by bath immersion. In the present study, we used a droplet digital PCR assay, a more sensitive and robust PCR method, to further investigate seawater transmission and shedding of ISAV. A comparison of the performance of the five different concentration methods showed that the use of the method of applying the electronegatively charged filter together with NucliSENS^®^ lysis buffer for elution resulted in detection of significantly higher levels of ISAV RNA from seawater by RT-ddPCR compared to the other four methods. The higher ISAV RNA concentration detected from seawater with the MF^-^/buffer 1 method (A) correlates with results from an in vitro study carried out with seawater samples spiked with ISAV [20], as well as from recent equivalent studies with salmonid alphavirus in seawater [20,26].

Given that, environmental samples are often associated with inhibitors, ISAV RNA from the seawater samples were analyzed by RT-ddPCR. Droplet digital PCR has been applied in water quality assessment [32,33,34,35], and several studies have found it to be superior to RT-qPCR in terms of sensitivity, specificity, and accuracy, as well as reproducibility [32,33,34,35]. In this study, we demonstrated that ISAV can be detected in seawater, after concentration of seawater samples.

The effect of viral dose on within-host infection dynamics in vivo is not completely understood in fish, but some general principles have emerged. Viral dose impacts the virus replication and the strength of the host-immune response [36,37]. In viruses that cause acute infections, such as influenza viruses, (which belong to Orthomyxoviridae family like ISAV), there is an inverse relationship between the size of viral dose and the infectious period, a critical determinant of transmission probability [38,39,40]. Data also indicate that the dose has an influence on the dynamics of immunity, and that the relationship is complex and nonlinear. For example, the level of antibodies developed after clearing an infection increases with a very large viral dose [40,41], but a dose of moderate size tends to produce an amount of antibodies comparable to that produced from a dose of small size. Thus, viral dose can influence both central component of viral fitness, viral transmission, and a major source of selection, host immunity, all of which is critical information needed for surveillance and detection of waterborne pathogens. In this study, bath challenge in seawater was used because it most closely mimics natural exposure to a pathogen [42]. Two different ISAV doses used in the bath challenge were selected based on previous experience with ISAV bath challenge experiments [31]. Both doses were able to cause infection in the challenged fish groups, as shown by detection of ISAV in gill and heart tissues. These results clearly demonstrate the highly infectious nature of the virus via horizontal transmission, through the water. Higher levels of ISAV RNA were detected in the seawater samples, gills, and heart tissues of high dose exposed fish compared to the low dose exposed fish. In the high dose fish, virus shedding in water peaked at 11 DPC (2.7 × 10^5^), one day before mortalities started, and at 16 DPC, all the fish were dead. However, in the low dose group, there were no mortalities until five days after virus shedding in water peaked at 12 DPC (1.6 × 10^5^). There were more than five days between the onset of mortality in high and low dose groups, but only one day between the peak of virus shedding in water. The mortality rate and the infection pattern observed in the low dose fish (Figure 4B) indicate this dose is approaching the minimum infective dose for the establishment of infection in seawater salmon populations. These findings are significant because they give indication of the relationship between ISAV infective dose, virus shedding in seawater salmon populations, and the on-set of mortality.

In this study, conclusions regarding sensitivity of the seawater filtration method, compared to sampling fish for virus detection in tissues, cannot be drawn. However, we recorded very high fish mortality and all seawater samples collected from challenge tanks came out positive for virus detection with the most sensitive filtration method. ISAV prevalence in seawater tanks and in the gill and heart samples at the peak of infection followed the same progression. In our previous laboratory experiments, limit of detection (LOD) of virus for the concentration method A was calculated to be 1.1 × 10^4^ ISAV RNA copies/L of natural seawater [20], which is similar to the average virus detection found in the present study. Nevertheless, in field condition, reliable detection of ISAV, which may be present in the seawater at very low concentrations, may require some further optimization of the method. It is plausible to note that several factors may affect ISAV detection in field environment. When one or more net pens are infected and virus shedding is ongoing, water currents and survival of the virus in the environment may influence disease transmission. Free virus, or virus associated with the lipid fraction released from dead or infected fish, may be passively transported by the water currents [43,44]. The virus concentration at the receiving farm may depend on the concentration of virus shed from infected sites combined with water currents, which might concentrate or dilute the virus particles, and this may affect the sensitivity of the method under field conditions. During the experimental design of the study, we simulated a natural virus transmission route via bath immersion, followed by a constant high water flow rate (mimicking high ocean current) in all tanks 950 L h-1. This allowed us to investigate disease transmission dynamics in more realistic manner under controlled laboratory conditions and quantify ISAV shedding in seawater.

Under field conditions, if infection prevalence in a farm is low, one must sample a high number of fish to ensure collection of at least one infected fish, which may lead to low sensitivity for the classical surveillance method. This strategy also has animal welfare and economical concerns, when clinically healthy fish are euthanized for surveillance purposes. If virus surveillance is based on seawater samples, live fish will not have to be killed, and the time, resources, and cost for sampling and analysis will be significantly reduced. It is important to note that the concentration method described here has also been used successfully to concentrate SAV shedding in a challenge trial [19] and in the field (Bernhardt et al., 2021 in press).

The water concentration method will be followed up with sampling of seawater in farms with ongoing outbreaks of ISA, in order to study the applicability of the method under practical conditions. In the field, environmental variables, such as sunlight and plankton predation [45], feces, mucus, aquatic parasites, and farming equipment [46,47] are considered key risk factors in the epidemiology of ISA [48]. Such factors may also have impact on the sensitivity of the water filtration method. Thus, our ability to detect ISAV from seawater collected from Atlantic salmon farms holding infected fish should be studied in the field to validate sensitivity and recovery efficiency of the method.

## 5. Conclusions

The current study confirmed that ISAV shed from infected fish in seawater can be concentrated from seawater samples by filtration and detected by using RT-ddPCR, which has previously been found to be sensitive and robust for evaluating environmental samples that are most often associated with PCR inhibitors. The method combining an electronegative membrane filter and NucliSENS^®^ lysis buffer was the best for ISAV concentration and detection in this study. The main route of ISAV is horizontal transmission in seawater. Thus, the development of a reliable method for the detection of ISA-virus in seawater can constitute a key to the implementation of preventive strategies to control disease, such as surveillance, as well as to assess the impact of ISA disease dynamics including risk of water borne virus transmission within and between farms.

## Figures and Tables

**Table 1:** Post hoc Tukey HSD pairwise comparisons of five concentration methods (A–E), the two membrane filters (MF^-^ or MD^+^) and the four buffer solutions (buffer 1–4). The columns indicate the null hypothesis, the mean difference, and the *p*-values. Negative mean differences in estimates in the first five rows indicate that method A recovered more copies of the ISAV genome than any of the other methods. All estimates are significant.

**Tukey Hypotheses for the Concentration Methods in High Dose Tanks**	**Mean Difference of Log (Copy Numbers)**	***p*** **-Value**
B–A = 0	−2.43	*p* < 0.001
C–A = 0	−2.15	*p* < 0.001
D–A = 0	−5.45	*p* < 0.001
E–A = 0	−5.94	*p* < 0.001
C–B = 0	0.28	*p* < 0.001
D–B = 0	−3.02	*p* < 0.001
E–B = 0	−3.51	*p* < 0.001
D–C = 0	−3.30	*p* < 0.001
E–C = 0	−3.80	*p* < 0.001
E–D = 0	−0.49	*p* < 0.001
**Tukey Hypotheses for Filter and Buffer** **(Model 2)**	**Mean Difference of Log** **(Copy Numbers)**	***p*-Value**
MF[–]–MD[+] (filters)	2.4	*p* < 0.001
Buffer 2–Buffer 1 = 0	−2.15	*p* < 0.001
Buffer 3–Buffer 1 = 0	−5.45	*p* < 0.001
Buffer 4–Buffer 1 = 0	−3.51	*p* < 0.001
Buffer 3–Buffer 2 = 0	−3.30	*p* < 0.001
Buffer 4–Buffer 2 = 0	−1.36	*p* < 0.001
Buffer 4–Buffer 3 = 0	1.95	*p* < 0.001

## Data Availability

Raw data generated during the study can be found at figshare.com (accessed on 9 July 2021): 10.6084/m9.figshare.14938608.

## References

[1] Aldrin M., Lyngstad T.M., Kristoffersen A.B., Storvik B., Borgan Ø., Jansen P.A. (2011). Modelling the spread of infectious salmon anaemia among salmon farms based on seaway distances between farms and genetic relationships between infectious salmon anaemia virus isolates. J. R. Soc. Interface.

[2] Gustafson L., Ellis S., Beattie M., Chang B., Dickey D., Robinson T., Marenghi F., Moffett P., Page F. (2007). Hydrographics and the timing of infectious salmon anemia outbreaks among Atlantic salmon (*Salmo salar* L.) farms in the Quoddy region of Maine, USA and New Brunswick, Canada. Prev. Vet. Med..

[3] Mardones F., Perez A., Carpenter T. (2009). Epidemiologic investigation of the re-emergence of infectious salmon anemia virus in Chile. Dis. Aquat. Org..

[4] Lyngstad T., Hjortaas M., Kristoffersen A., Markussen T., Karlsen E., Jonassen C., Jansen P. (2011). Use of Molecular Epidemiology to Trace Transmission Pathways for Infectious Salmon Anaemia Virus (ISAV) in Norwegian Salmon Farming. Epidemics.

[5] Cunningham C.O., Gregory A., Black J., Simpson I., Raynard R.S. (2002). A novel variant of the infectious salmon anaemia virus (ISAV) haemagglutinin gene suggests mechanisms for virus diversity. Bull. Eur. Assoc. Fish Pathol. B Eur. Assoc. Fish Pat..

[6] Mjaaland S., Hungnes O., Teig A., Dannevig B.H., Thorud K., Rimstad E. (2002). Polymorphism in the Infectious Salmon Anemia Virus Hemagglutinin Gene: Importance and Possible Implications for Evolution and Ecology of Infectious Salmon Anemia Disease. Virology.

[7] Nylund A., Devold M., Plarre H., Isdal E., Aarseth M. (2003). Emergence and maintenance of infectious salmon anaemia virus (ISAV) in Europe: A new hypothesis. Dis. Aquat. Org..

[8] Christiansen D.H., Østergaard P.S., Snow M., Dale O.B., Falk K. (2010). A low-pathogenic variant of infectious salmon anemia virus (ISAV-HPR0) is highly prevalent and causes a non-clinical transient infection in farmed Atlantic salmon (*Salmo salar* L.) in the Faroe Islands. J. Gen. Virol..

[9] Kibenge F.S., Godoy M., Wang Y., Kibenge M.J., Gherardelli V., Mansilla S., Lisperger A., Jarpa M., Larroquete G., Avendaño F. (2009). Infectious salmon anaemia virus (ISAV) isolated from the ISA disease outbreaks in Chile diverged from ISAV isolates from Norway around 1996 and was disseminated around 2005, based on surface glycoprotein gene sequences. Virol. J..

[10] Krossøy B., Devold M., Sanders L., Knappskog P.M., Aspehaug V., Falk K., Nylund A., Koumans S., Endresen C., Biering E. (2001). Cloning and identification of the infectious salmon anaemia virus haemagglutininThe GenBank accession numbers of the sequences reported in this study are AF302799–AF302803 and AF309075. J. Gen. Virol..

[11] Falk K., Aspehaug V., Vlasak R., Endresen C. (2004). Identification and Characterization of Viral Structural Proteins of Infectious Salmon Anemia Virus. J. Virol..

[12] Aspehaug V., Mikalsen A.B., Snow M., Biering E., Villoing S. (2005). Characterization of the Infectious Salmon Anemia Virus Fusion Protein. J. Virol..

[13] Aamelfot M., Christiansen D.H., Dale O.B., McBeath A., Benestad S.L., Falk K. (2016). Localised Infection of Atlantic Salmon Epithelial Cells by HPR0 Infectious Salmon Anaemia Virus. PLoS ONE.

[14] Aamelfot M., Dale O.B., Falk K. (2014). Infectious salmon anaemia—Pathogenesis and tropism. J. Fish Dis..

[15] Rimstad E., Dale O.B., Dannevig B.H., Falk K. (2011). Infectious salmon anaemia. J. Fish Dis..

[16] Hjeltnes B. (2018). Fish Health Report 2018.

[17] Lyngstad T., Jansen P., Sindre H., Jonassen C., Hjortaas M., Johnsen S., Brun E. (2008). Epidemiological investigation of infectious salmon anaemia (ISA) outbreaks in Norway 2003–2005. Prev. Vet. Med..

[18] Qviller L., Kristoffersen A.B., Lyngstad T.M., Lillehaug A. (2020). Infectious Salmon Anemia and Farm-Level Culling Strategies. Front. Veter Sci..

[19] Bernhardt L.-V., Myrmel M., Lillehaug A., Qviller L., Weli S.C. (2021). Concentration and detection of salmonid alphavirus in seawater during a post-smolt salmon (Salmo salar) cohabitant challenge. Dis. Aquat. Org..

[20] Weli S.C., Tartor H., Spilsberg B., Dale O.B., Lillehaug A. (2021). Short communication: Evaluation of charged membrane filters and buffers for concentration and recovery of infectious salmon anaemia virus in seawater. PLoS ONE.

[21] Dannevig B.H., Falk K., Press C.M. (1995). Propagation of infectious salmon anaemia (ISA) virus in cell culture. Veter. Res..

[22] Devold M., Krossøy B., Aspehaug V., Nylund A. (2000). Use of RT-PCR for diagnosis of infectious salmon anaemia virus (ISAV) in carrier sea trout Salmo trutta after experimental infection. Dis. Aquat. Org..

[23] Reed L., Muench H. (1938). A SIMPLE METHOD OF ESTIMATING FIFTY PER CENT ENDPOINTS12. Am. J. Epidemiol..

[24] Weli S.C., Aamelfot M., Dale O.B., Koppang E.O., Falk K. (2013). Infectious salmon anaemia virus infection of Atlantic salmon gill epithelial cells. Virol. J..

[25] Aspehaug V., Falk K., Krossøy B., Thevarajan J., Sanders L., Moore L., Endresen C., Biering E. (2004). Infectious salmon anemia virus (ISAV) genomic segment 3 encodes the viral nucleoprotein (NP), an RNA-binding protein with two monopartite nuclear localization signals (NLS). Virus Res..

[26] Weli S.C., Bernhardt L.-V., Qviller L., Myrmel M., Lillehaug A. (2021). Development and evaluation of a method for concentration and detection of salmonid alphavirus from seawater. J. Virol. Methods.

[27] Snow M., McKay P., A McBeath A.J., Black J., Doig F., Kerr R., Cunningham C.O., Nylund A., Devold M. (2006). Development, application and validation of a Taqman real-time RT-PCR assay for the detection of infectious salmon anaemia virus (ISAV) in Atlantic salmon (Salmo salar). Dev. Boil..

[28] R Core Team (2014). R: A Language and Environment for Statistical Computing (Version 3.1.2).

[29] Hothorn T., Bretz F., Westfall P. (2008). Simultaneous Inference in General Parametric Models. Biom. J..

[30] Kleiber C., Zeileis A. (2008). Applied Econometrics with R.

[31] Aamelfot M., Dale O.B., Weli S.C., Koppang E.O., Falk K. (2012). Expression of the Infectious Salmon Anemia Virus Receptor on Atlantic Salmon Endothelial Cells Correlates with the Cell Tropism of the Virus. J. Virol..

[32] Rački N., Morisset D., Gutierrez-Aguirre I., Ravnikar M. (2014). One-step RT-droplet digital PCR: A breakthrough in the quantification of waterborne RNA viruses. Anal. Bioanal. Chem..

[33] Cao Y., Raith M.R., Griffith J.F. (2015). Droplet digital PCR for simultaneous quantification of general and human-associated fecal indicators for water quality assessment. Water Res..

[34] Te S.H., Chen E.Y., Gin K.Y.-H. (2015). Comparison of Quantitative PCR and Droplet Digital PCR Multiplex Assays for Two Genera of Bloom-Forming Cyanobacteria, Cylindrospermopsis and Microcystis. Appl. Environ. Microbiol..

[35] Nshimyimana J.P., Martin S.L., Flood M., Verhougstraete M.P., Hyndman D.W., Rose J.B. (2018). Regional Variations of Bovine and Porcine Fecal Pollution as a Function of Landscape, Nutrient, and Hydrological Factors. J. Environ. Qual..

[36] Jarungsriapisit J., Moore L.J., Mæhle S., Skår C., Einen A.C., Fiksdal I.U., Morton H.C., Stefansson S.O., Taranger G.L., Patel S. (2016). Relationship between viral dose and outcome of infection in Atlantic salmon, Salmo salar L., post-smolts bath-challenged with salmonid alphavirus subtype 3. Veter. Res..

[37] Moore L., Jarungsriapisit J., Nilsen T., Stefansson S., Taranger G., Secombes C., Morton H.C., Patel S. (2017). Immune gene profiles in Atlantic salmon (salmo salar L.) post-smolts infected with SAV3 by bath-challenge show a delayed response and lower levels of gene transcription compared to injected fish. Fish Shellfish Immunol..

[38] Larson E.W., Dominik J.W., Rowberg A.H., Higbee G.A. (1976). Influenza virus population dynamics in the respiratory tract of experimentally infected mice. Infect. Immun..

[39] Ottolini M.G., Blanco J., Eichelberger M.C., Porter D.D., Pletneva L., Richardson J.Y., Prince G.A. (2005). The cotton rat provides a useful small-animal model for the study of influenza virus pathogenesis. J. Gen. Virol..

[40] Ottolini M.G., Porter D.D., Hemming V.G., Hensen S.A., Sami I.R., Prince G.A. (1996). Semi-permissive replication and functional aspects of the immune response in a cotton rat model of human parainfluenza virus type 3 infection. J. Gen. Virol..

[41] Wherry E.J., McElhaugh M.J., Eisenlohr L.C. (2002). Generation of CD8+T Cell Memory in Response to Low, High, and Excessive Levels of Epitope. J. Immunol..

[42] Nordmo R. (1997). Strengths and weaknesses of different challenge methods. Dev. Boil. Stand..

[43] Kristoffersen A., Viljugrein H., Kongtorp R., Brun E., Jansen P. (2009). Risk factors for pancreas disease (PD) outbreaks in farmed Atlantic salmon and rainbow trout in Norway during 2003–2007. Prev. Vet. Med..

[44] Stene A., Hellebø A., Viljugrein H., Solevåg S., Devold M., Aspehaug V. (2016). Liquid fat, a potential abiotic vector for horizontal transmission of salmonid alphavirus?. J. Fish Dis..

[45] Middelboe M., Brussaard C.P.D. (2017). Marine Viruses: Key Players in Marine Ecosystems. Viruses.

[46] Nylund A., Hovland T., Hodneland K., Nilsen F., Lovik P. (1994). Mechanisms for transmission of infectious salmon anaemia (ISA). Dis. Aquat. Org..

[47] Valdes-Donoso P., Mardones F., Jarpa M., Ulloa M., Carpenter T., Perez A. (2013). Co-infection patterns of infectious salmon anaemia and sea lice in farmed A tlantic salmon, *Salmo salar* L., in southern C hile (2007–2009). J. Fish Dis..

[48] Gustafson L., Ellis S., Bartlett C. (2005). Using expert opinion to identify risk factors important to infectious salmon-anemia (ISA) outbreaks on salmon farms in Maine, USA and New Brunswick, Canada. Prev. Vet. Med..

